# Cryptogenic Multifocal Infarctions Involving Brain and Spinal Cord in a Patient With Patent Foramen Ovale: A Case Report

**DOI:** 10.1155/crnm/9962894

**Published:** 2026-08-19

**Authors:** Mohamed Zuhail Kizhakka Peediyakkal, Nabil Sherif Mahmood, Ashraf Abdulla Molokhia, Bara Mahmoud F. Alqudah, Navaf K. Mohamedali, Georgy Itty Paniker, Ahmed Daniyal Nawaz, Abdulqadir Nashwan

**Affiliations:** ^1^ Critical Care Medicine, Hamad Medical Corporation, Doha, Qatar, hamad.qa; ^2^ Clinical Imaging, Hamad Medical Corporation, Doha, Qatar, hamad.qa; ^3^ Medicine, Hamad Medical Corporation, Doha, Qatar, hamad.qa; ^4^ Nursing and Midwifery Research, Hamad Medical Corporation, Doha, Qatar, hamad.qa

## Abstract

We report the case of a 46‐year‐old female with a history of gestational diabetes mellitus (GDM) who presented with acute onset flaccid quadriplegia, respiratory distress, and multifocal cerebral infarctions. Initial clinical suspicion favored inflammatory myelopathy; however, advanced neuroimaging demonstrated a long‐segment anterior spinal cord infarction extending from the cervical to upper thoracic region, along with bilateral cerebellar and occipitoparietal infarcts. A patent foramen ovale and hypoplastic vertebral artery suggested a possible embolic and hemodynamic mechanism. This case illustrates the diagnostic complexity in distinguishing vascular infarction from inflammatory myelopathies and underscores the importance of thorough imaging and vascular workup in acute flaccid paralysis.

## 1. Introduction

Acute flaccid quadriplegia is a medical emergency with a wide differential diagnosis, including transverse myelitis, Guillain–Barré syndrome (GBS), spinal cord infarction, and demyelinating diseases such as neuromyelitis optica spectrum disorder (NMOSD) and multiple sclerosis (MS) [1]. Among these, anterior spinal cord infarction (ASCI) presents a diagnostic challenge due to overlapping clinical and radiological features with inflammatory myelopathies [2]. Early differentiation is essential, as the underlying mechanisms, treatment strategies, and outcomes differ significantly.

## 2. Case Presentation

A 46‐year‐old woman with a history of gestational diabetes mellitus, managed with metformin, presented to the emergency department with severe pain in the lower neck and upper back. Within 24 h prior to presentation, she developed rapidly progressive weakness that began in the right upper limb, followed by the left upper limb, and then involved both lower limbs. Sensation remained preserved, with no bowel or bladder dysfunction. She later developed dysarthria, shallow breathing, and reduced speech capacity.

On examination, she had flaccid quadriplegia, areflexia, and respiratory distress with a forced vital capacity of 15% predicted. Initial laboratory investigations showed mild metabolic acidosis, with otherwise unremarkable hematological and biochemical parameters (Tables 1 and 2). Initial CT of the head and cervical spine was unremarkable. Due to rapid deterioration, she was intubated and admitted to the MICU. Initial working diagnosis included inflammatory myelopathy, and one dose of immunoglobulin was administered.

**TABLE 1:** Blood chemistry panel.

Parameter	Result	Reference range	Comments
Sodium	134 mmol/L	133–146 mmol/L	Normal
Potassium	4 mmol/L	3.5–5.3 mmol/L	Sample hemolyzed
Chloride	101 mmol/L	95–108 mmol/L	Normal
Bicarbonate	17 mmol/L	22–29 mmol/L	Low (metabolic acidosis)
Adjusted calcium	2.35 mmol/L	2.20–2.60 mmol/L	Normal
Magnesium	0.79 mmol/L	0.70–1.00 mmol/L	Normal
Glucose	8.0 mmol/L	3.3–5.5 mmol/L	High
CRP	1.4 mg/L	0.0–5.0 mg/L	Normal

**TABLE 2:** Hematology panel.

Hemoglobin (Hgb)	13.1 g/dL	12.0–15.0 g/dL	Normal
Hematocrit (Hct)	39.50%	36.0%–46.0%	Normal
Mean corpuscular volume (MCV)	93.8 fL	83.0–101.0 fL	Normal
Platelet count	256 × 10^3^/μL	150–410 × 10^3^/μL	Normal
Prothrombin time (PT)	11.8 s	9.4–12.5 s	Normal
International normalized ratio (INR)	1	0.9–1.1	Normal

**FIGURE 1:**
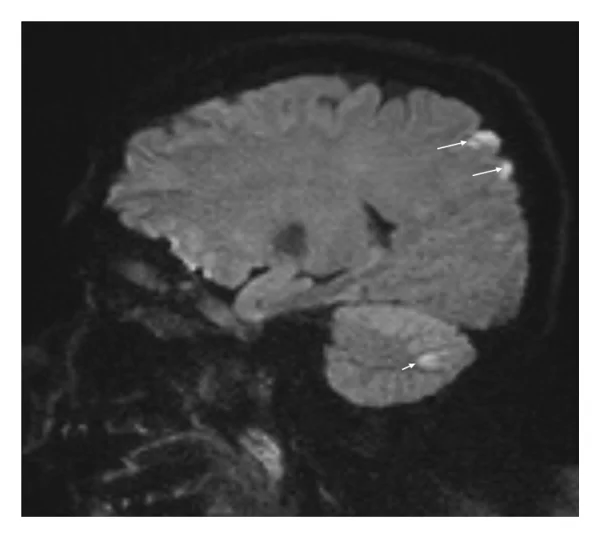
Sagittal diffusion‐weighted (DW) image of the brain demonstrating several small cortical‐based high signal findings in the parietal region (long white arrows) and the left cerebellar hemisphere (short white arrow) in keeping with multiple small infarcts.

**FIGURE 2:**
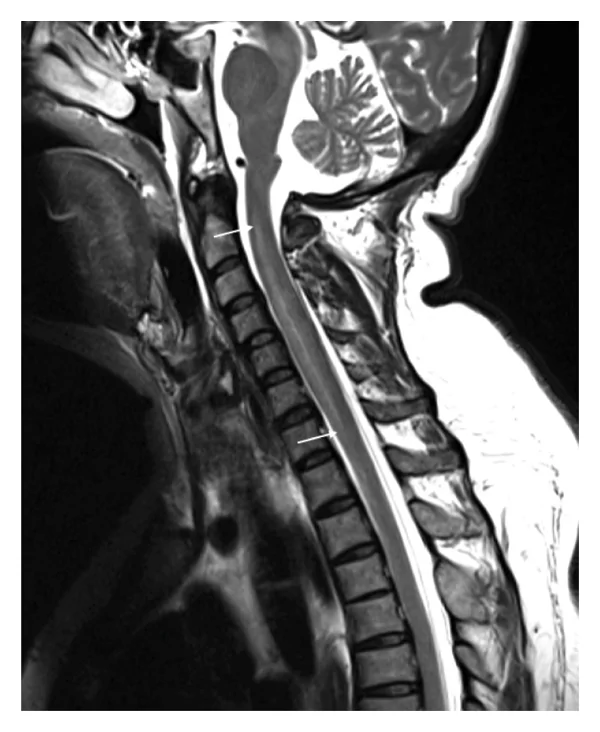
Sagittal T2W image of the cervical spine demonstrating high T2W intramedullary signal intensity involving the anterior cervical spinal cord with mild cord swelling (upper and lower margins marked by white arrows) in keeping with an acute infarct.

**FIGURE 3:**
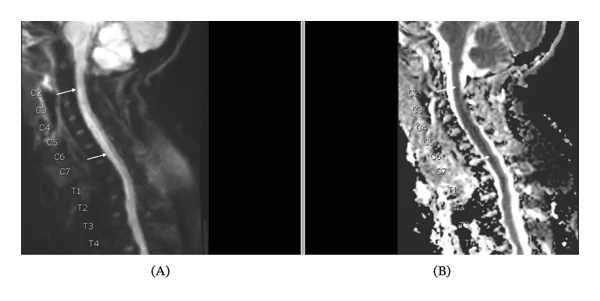
Sagittal DWI (A) and ADC (B) image of the cervical spine demonstrating high DW signal intensity involving the anterior cervical spinal cord (white arrows in A) appearing dark on ADC (white arrows in B) in keeping with an acute infarct.

**FIGURE 4:**
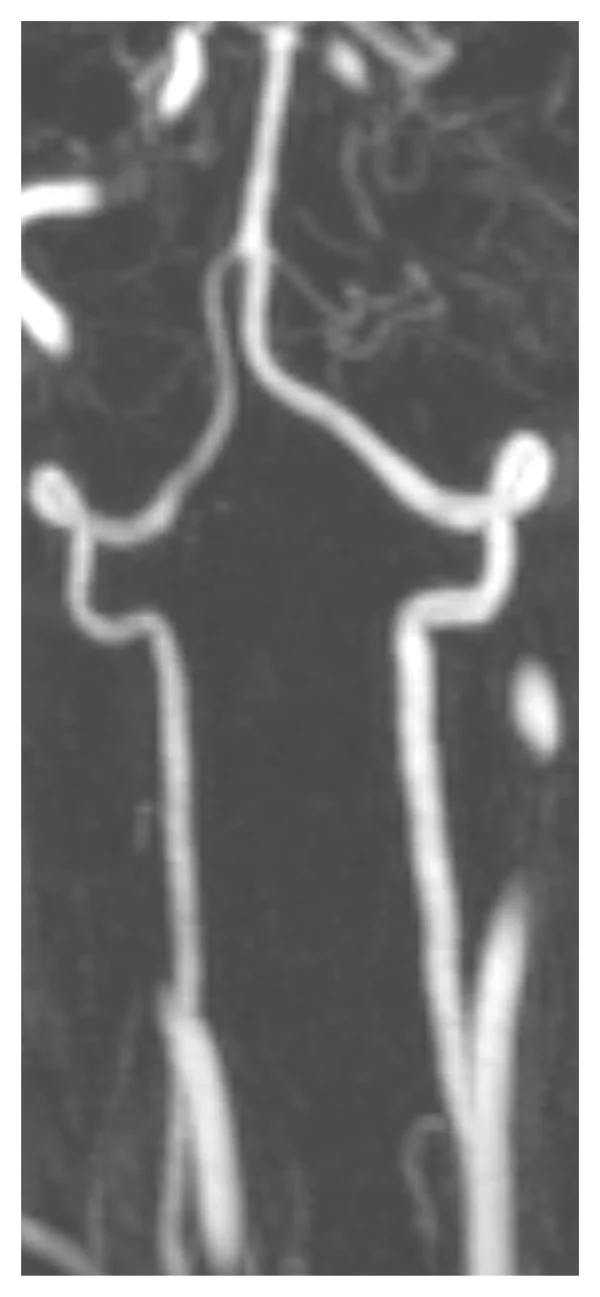
Coronal MIP image from an MRA study of the neck demonstrating diffuse narrowing of the right vertebral artery as compared to the left in keeping with a hypoplastic right vertebral artery.

MRI brain revealed multifocal acute infarctions involving bilateral cerebellar and occipitoparietal regions (Figure 1). MRI spine demonstrated long‐segment anterior spinal cord diffusion restriction from C2 to T3, consistent with ASCI (Figures 2 and 3). MRA showed hypoplastic right vertebral artery (Figure 4). Echocardiography which was done as a part of evaluation identified a small patent foramen ovale raising the possibility of a paradoxical embolic event.

CSF analysis was normal with no pleocytosis or oligoclonal bands, effectively excluding inflammatory demyelinating disease. Autoimmune and thrombophilia screening were unremarkable except for mildly reduced protein S activity (Tables 3 and 4). Her screening duplex ultrasonography showed normal bilateral lower limb venous systems.

**TABLE 3:** Immunology and autoimmune panel.

Test	Result	Reference range	Interpretation
Serum IgA	4.28 ⟶ 3.87 g/L	0.70–4.00 g/L	Initially high, then normalized
Serum IgG	20.48 g/L	6.10–16.16 g/L	Elevated
Antinuclear antibody (ANA)	Positive (1:40)	Negative	Low titer, nonspecific
Anti‐dsDNA	Negative	Negative	Negative
Protein S activity	52.40%	56.1%–126.0%	Mildly low
Protein C activity	85.60%	70.0%–140.0%	Normal

**TABLE 4:** Cerebrospinal fluid (CSF) analysis.

Test	Result	Reference range	Interpretation
Appearance	Clear	Clear	Normal
Total nucleated cells	0 cells/μL	0–5 cells/μL	Normal
Red blood cells (RBC)	3 cells/μL	—	Minimal contamination
Protein	0.19 g/L	0.15–0.45 g/L	Normal
Glucose	4.46 mmol/L	2.22–3.89 mmol/L	Mildly elevated
CSF IgG	27 mg/L	0–34 mg/L	Normal
IgG Index	0.5	0.3–0.6	Normal
Oligoclonal bands	Negative	Negative	No intrathecal IgG synthesis

Together, the laboratory and imaging findings supported a diagnosis of ASCI accompanied by multiple acute cerebral infarcts, with no infectious, inflammatory, or autoimmune cause identified. The patient underwent tracheostomy due to prolonged ventilatory requirements, following which antiplatelet therapy was initiated after exclusion of hemorrhagic and inflammatory etiologies. She was managed with a comprehensive critical care approach, including lung protective mechanical ventilation with gradual weaning protocols, pharmacological and mechanical prophylaxis for deep vein thrombosis, meticulous nutritional optimization tailored to her metabolic demands, and strict prevention of ICU‐related complications such as ventilator associated pneumonia, pressure injuries, and critical illness neuropathy.

Early, structured multidisciplinary rehabilitation was initiated, incorporating intensive respiratory physiotherapy with airway clearance techniques and inspiratory muscle training, alongside progressive passive and active‐assisted limb mobilization to prevent contractures and muscle atrophy. This was followed by graded strengthening exercises, neuromuscular re‐education, and functional training aimed at improving motor recovery and respiratory independence.

Over the subsequent weeks, the patient demonstrated gradual improvement in respiratory mechanics and neuromuscular function. She was subsequently transferred to a specialized rehabilitation facility for continued comprehensive neurorehabilitation and functional recovery.

## 3. Discussion

This case is unique due to the simultaneous occurrence of extensive ASCI and multifocal posterior circulation cerebral infarctions in a relatively young patient, initially mimicking inflammatory myelopathy. The coexistence of long‐segment cervical spinal cord ischemia, bilateral cerebellar infarcts, and occipitoparietal cortical strokes, in the absence of major atherosclerotic disease or systemic vasculitis, is exceedingly rare and sparsely described in the literature.

Spinal cord infarction accounts for less than 1% of all strokes, with anterior spinal artery infarction being the most common subtype. Concurrent spinal cord and multifocal cerebral infarctions are exceptionally rare. Distinguishing ASCI from inflammatory myelopathies is critical. Demyelinating disorders typically present with subacute progression, sensory involvement, and CSF abnormalities whereas ASCI presents abruptly with axial pain, preserved dorsal column function, and diffusion restriction on MRI confined to vascular territories [2]. The acute onset, neck pain, and rapid progression to quadriplegia in our patient raised suspicion for an inflammatory myelopathy. However, normal CSF and the absence of systemic autoimmune markers made transverse myelitis and NMOSD unlikely.

This case likely represents a multifactorial vascular event. The presence of a patent foramen ovale provides a potential pathway for paradoxical embolism, allowing venous thrombi to bypass pulmonary filtration and enter systemic circulation. While the clinical relevance of small PFOs remains debated, numerous studies have highlighted their association with cryptogenic stroke, particularly in younger individuals without clear vascular risk factors [3, 4]. Although no thrombus or other high‐risk cardiac embolic source was identified, the PFO remains a plausible contributor to the multifocal infarcts seen in this case [5]. Additionally, the hypoplastic vertebral artery may have contributed to compromised posterior circulation and reduced perfusion of the anterior spinal artery [6]. The combination of embolic phenomena and pre‐existing vascular insufficiency likely predisposed the spinal cord to ischemia. This dual mechanism highlights the importance of evaluating both embolic sources and vascular anatomy in cryptogenic infarction [7]. No standardized treatment protocols exist for spinal cord infarction due to its rarity, and therapy remains largely supportive [8]. Recent interest has emerged around the potential use of neuroprotective strategies and rehabilitation‐driven functional recovery.

Prognosis in spinal cord infarction is generally poor, especially with cervical involvement. Functional recovery depends on the extent and level of injury. Our patient showed gradual improvement in ventilator weaning over weeks, highlighting the role of intensive rehabilitation. Emerging therapies are under investigation, particularly in the area of neuroregeneration and rehabilitation. Preclinical studies have explored mesenchymal stem cell transplantation, epidural electrical stimulation, and neurotrophic factors as adjuncts to improve motor recovery, though these remain experimental [9]. Functional electrical stimulation (FES) and robotic‐assisted rehabilitation devices have shown some promise in improving outcomes in incomplete spinal cord injuries and may be considered in long‐term care planning [10].

## 4. Conclusion

This case highlights an uncommon presentation of concurrent anterior spinal cord and multifocal cerebral infarctions manifesting as acute flaccid quadriplegia with respiratory compromise. The diagnostic challenge lies in its close resemblance to inflammatory myelopathies; however, early diffusion‐weighted MRI of both brain and spinal cord was pivotal in establishing an ischemic etiology and avoiding inappropriate immunotherapy. The coexistence of a patent foramen ovale and vertebral artery hypoplasia suggests a multifactorial mechanism involving paradoxical embolism and regional hypoperfusion.

From a clinical perspective, this case underscores the need to consider vascular causes in acute nontraumatic paralysis and to pursue timely multimodal imaging and cardioembolic evaluation. Although therapeutic options remain largely supportive, early critical care optimization and structured neurorehabilitation can significantly influence functional recovery. Further research is warranted to better define mechanisms and guide targeted management strategies in such rare but clinically significant presentations.

## Author Contributions

Mohamed Zuhail Kizhakka Peediyakkal: manuscript writing and concept.

Nabil Sherif Mahmood: radiological interpretation.

Ashraf Abdulla Molokhia: manuscript final revision and correction.

Bara Mahmoud F. Alqudah: manuscript writing.

Navaf K. Mohamedali and Georgy Itty Paniker: literature review.

Ahmed Daniyal Nawaz: references.

Abdulqadir Nashwan: manuscript revision.

## Funding

The authors received no financial support for the research, authorship, or publication of this article.

## Consent

The authors confirm that written informed consent was obtained from the patient’s legal representative for the publication of this case report and the associated images.

## Conflicts of Interest

The authors declare no conflicts of interest.

## Data Availability

The clinical and radiological data used to support the findings of this study are available from the corresponding author upon request.
